# Near-Full-Length Genome Sequence of a Novel Reovirus from the Chinese Mitten Crab, *Eriocheir sinensis*

**DOI:** 10.1128/genomeA.00447-15

**Published:** 2015-05-21

**Authors:** Huaishun Shen, Yuanchao Ma, Yacheng Hu

**Affiliations:** a Freshwater Fisheries Research Center, Key Laboratory of Freshwater Fisheries and Germplasm Resources Utilization, Ministry of Agriculture, Chinese Academy of Fishery Sciences, Wuxi, China; b Wuxi Fisheries College, Nanjing Agricultural University, Nanjing, China

## Abstract

A novel *Eriocheir sinensis* reovirus (EsRV) was identified using deep-sequencing techniques in crabs afflicted with trembling disease (TD). Near-full-length genome sequences of 12 segments of EsRV were obtained. The genome of EsRV will facilitate further studies on the causative agent of TD.

## GENOME ANNOUNCEMENT

The freshwater Chinese mitten crab, *Eriocheir sinensis*, is one of the most economically important indigenous crustaceans in freshwater aquaculture production in China (1). Trembling disease (TD) of *E. sinensis* was first reported in culture ponds in Jiangsu Province, China, in 1994 (2). Crabs with TD exhibit trembling of the legs, sluggishness, and loss of appetite. TD has caused severe economic losses in recent years, as the mortality of crabs with TD reaches 70%, but the etiological agent of TD has not been confirmed (3).

Reovirus, a double-stranded RNA virus that has 9 to 12 linear genome segments, has been found in many host species, including vertebrate and invertebrate animals and plants (4–7). To date, only one full-length genome sequence of a crab-originating reovirus (*Scylla serrata* reovirus SZ-2007 [SsRV]) has been sequenced (7–9). Here, we report the near-full-length genome sequencing using a deep-sequencing approach of a novel reovirus obtained from Chinese mitten crabs displaying signs of TD.

An RNA-sequencing (RNA-seq) library was prepared, according to the manufacturer’s protocol, and sequencing was performed on an Illumina HiSeq 2000 instrument. A total of 251 million raw reads were generated. These raw reads were assembled, and a total of 75,139 nonredundant genes were produced. All assembled nonredundant genes were used in a BLAST against genes in the nonredundant (nr) database. We found 12 gene sequences that were significantly similar to the full-genome sequences of *S. serrata* reovirus SZ-2007 (SsRV) (*E* value, <4.80e-86). The SsRV genome includes 12 double-stranded RNA (dsRNA) gene segments, suggesting that segments 1 to 12 compose the whole genome of a novel reovirus. Here, we named this novel reovirus *E. sinensis* reovirus WX-2012 (EsRV).

The total length of the 12 gene segments in EsRV was 23.913 kb, which is close to the length of the full genome of SsRV (24.464 kb). The identity of the total genome sequences between the two viruses is 69%. To confirm the completeness of the genome sequenced for EsRV, the 5′- and 3′-terminal conserved sequences were analyzed; reovirus genes have conserved 5′- and 3′-terminal sequences (7–9). Here, 5′ conserved terminal sequences were found in segments 3 to 8, 10, and 11, and 3′ conserved terminal sequences were found in 11 gene segments (all but segment 5). This result shows that we obtained full-length sequences of seven gene segments: segments 3, 4, 6 to 8, 10, and 11. The sequence alignment between EsRV and SsRV showed that three gene segments, 2, 9, and 12, were very near the 5′ end, but 5′ conserved terminal sequences were missed, suggesting that we obtained near-full-length sequences of these three genes. The sequence alignment between EsRV and SsRV also showed that we did not obtain the 5′ noncoding sequence of segment 1 and obtained only a partial 3′ noncoding sequence of segment 5. In summary, our results indicated that we obtained the near-full-length genome sequence of EsRV.

### Nucleotide sequence accession numbers. 

The genome sequence of the *E. sinensis* reovirus WX-2012 was submitted to GenBank with the accession numbers KP638402 to KP638413.
